# Effects of shuxuetong injection for cerebral infarction: A protocol for systematic review and meta-analysis

**DOI:** 10.1097/MD.0000000000021929

**Published:** 2020-08-28

**Authors:** Hua Fang, Honglong Zhou, Jicai Zhang, Ziyi Li, Zhen Chen, RaoRao Yuan, Xiangqun Huang, Junyong Yang, Jianqiang Zhang, Shuo Wang, Yong Huang, Shangwei Hu

**Affiliations:** a The Second Affiliated Hospital of Nanchang University, Nanchang; b Zhangshu City People's Hospital, Zhangshu; c Jiangxi Dexing Hospital, Dexing; d The Second People's Hospital of Jingdezhen, Jingdezhen, China; e Nanfeng People's Hospital, Nanfeng, China.

**Keywords:** cerebral infarction, meta-analysis, protocol, shuxuetong

## Abstract

**Background:**

Cerebral infarction (CI) is a common disease with high morbidity and disability. Shuxuetong (SXT) injection is a Chinese Materia Medica standardized product used in the treatment of CI. Currently, there is a lack of high-quality evidence to support the effectiveness and safety of SXT on patients with CI. This systematic review protocol aims at describing a meta-analysis to evaluate the efficacy of SXT for the treatment of CI.

**Methods:**

We will search the databases of PubMed, MEDLINE, Embase, Cochrane Library Central Register of Controlled Trials, China national knowledge infrastructure database (CNKI), Wan fang database, Chongqing VIP information, and SinoMed from their inception to Jun 2020. Two reviewers will independently screen Randomized controlled trials of SXT for the treatment of CI. The meta-analysis will be conducted using RevMan V.5.3 software.

**Results:**

The results of this study will be published in a peer-reviewed journal.

**Conclusion:**

The conclusion of our systematic review will provide evidence to judge whether SXT is an effective intervention for patients with CI.

**Trial registration number:**

10.17605/OSF.IO/3F6ZH.

## 1 Introduction

Cerebral infarction (CI) is an ischemic condition of the brain due to the anoxic and ischemic state after blood circulation is disrupted.^[1]^ It is a common disease with high morbidity and is ranked as the leading cause of disability around the world.^[2]^ Patients with CI often manifest as paralysis, difficulty swallowing, double vision or vision loss, confusion, vertigo, difficulty speaking or understanding speech.^[3]^ Moreover, CI brings significant psychological pressure and a huge economic burden to patients.^[4]^ Thus, effective treatment of CI is of great significance to alleviate the symptoms of the disease and improve the quality of life.

Shuxuetong (SXT) injection, a Chinese Materia Medica standardized product extracted from *Hirudo* and *Pheretima*, has been widely used in the treatment of CI and is reported to have good therapeutic effects in the clinic.^[5]^ Studies have shown that SXT has a protective effect on the brain tissue of ischemia-reperfusion and is effective for stroke recovery.^[6,7]^ Experiment in rats revealed that SXT can alleviate the injury of brain through regulating the PI3K-AKT and MAPK signaling pathways.^[8]^ In addition, its protective effect is related to reducing the production of mitochondrial superoxide, inhibiting inflammation, and inhibition of the vascular endothelial growth factor.^[9]^ Although numerous studies have demonstrated that SXT has good clinical effects on CI. There is no comprehensive and systematic evidence to confirm its clinical efficacy and safety. Thus, we will systematically compare the efficacy and safety of SXT in the treatment of CI, thereby provide a reference for clinical application.

## 2 Material and methods

This protocol is reported following the preferred reporting items for systematic reviews and meta-analysis protocols (PRISMA-P) statement guidelines.^[10]^ We have registered this study at Open Science Framework (OSF, https://osf.io/). The registration DOI of this study is 10.17605/OSF.IO/3F6ZH. If there are any changes, we will update the changes in our full review.

### 2.1 Inclusion criteria

#### 2.1.1 Study type

In this work, we will include randomized controlled trials (RCTs) of SXK of any size and duration in adult populations (≥18 years). Non-randomized control studies and observational study will be excluded. Studies published in English and Chinese will be included.

#### 2.1.2 Types of patients

This study will include patients diagnosed with CI by head computed tomography/ magnetic resonance imaging. Included patients had no restrictions on age, sex, economic status, severity of the disease, or education.

#### 2.1.3 Intervention type

Studies in which interventions involved SXK alone or combined with other routine pharmacotherapies will be included. In the control group, interventions will include placebo or conventional pharmacotherapies recommended by guidelines. Studies with different conventional pharmacotherapies in the control and treatment groups will be excluded.

#### 2.1.4 Outcomes

The criterion of therapeutic efficiency on neurological functions and daily living activities will be assessed by the National Institute of Health Stroke Scale (NIHSS), modified Rankin Scale (mRS), activities of daily living (ADL), and Barthel Index (BI). The cognitive functions will be evaluated by mini-mental state examination (MMSE) and Montreal Cognitive Assessment (MoCA).

### 2.2 Search strategy

Eligible studies in PubMed, MEDLINE, Embase, Cochrane Library Central Register of Controlled Trials, China national knowledge infrastructure database (CNKI), Wan fang database, Chongqing VIP information, and SinoMed will be searched by two authors from their inception to July 2020, independently. Moreover, relevant studies in Google scholar and Baidu Scholar will also be retrieved. The search strategy in Pubmed is as follows:

1#: Search: (((((((cerebral infarction[MeSH Terms]) OR (stroke[MeSH Terms])) OR (brain ischemia[MeSH Terms])) OR (hypoxia, brain[MeSH Terms])) OR (brain infarction[MeSH Terms])) OR (ischemic cerebral infarction[Title/Abstract])) OR (ischemic stroke[Title/Abstract])) OR (ischemic brain infarction[Title/Abstract]).
2#: Search: (shuxuetong[MeSH Terms]) OR (shuxuetong injection[Title/Abstract]).
3#: Search: (((((((((clinical trials, randomized[MeSH Terms]) OR (randomized controlled trial[MeSH Terms])) OR (controlled clinical trials, randomized[MeSH Terms])) OR (random allocation[MeSH Terms])) OR (RCT[Title/Abstract])) OR (controlled clinical trial[Title/Abstract])) OR (randomized[Title/Abstract])) OR (trial[Title/Abstract]).
#1 and #2 and #3

### 2.3 Data collection and analysis

#### 2.3.1 Selection of studies

**Figure 1:**
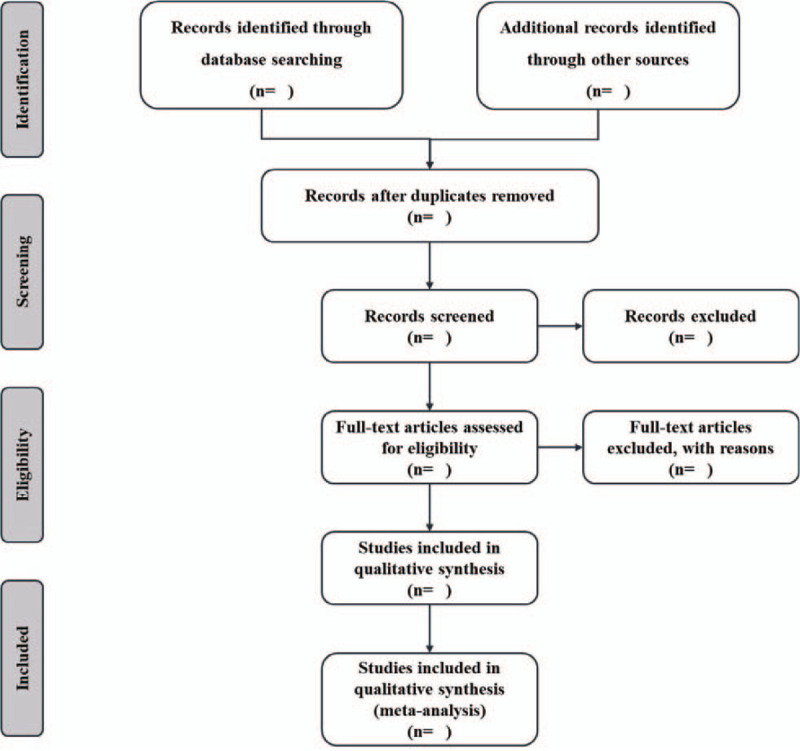
Flow chart of study selection.

The articles from the above databases will be exported by EndNote X9.0 (Stanford, Connecticut, https://endnote.com). Two reviewers will independently evaluate the titles and abstracts based on the research criteria. Next, the full versions will be accessed for inclusion by reading the full text. Any disagreements generated between the two reviewers will be resolved by consensus with other reviewers. The details of the selection process are shown in Figure 1.

#### 2.3.2 Data extraction

We will extract and record the first author's name, year of publication, study design, group information, age, gender, dropouts, sample size, duration of intervention, outcomes, and adverse effects from the studies that met the inclusion criteria. We will contact the corresponding authors for additional information if necessary.

#### 2.3.3 Risk of bias assessment

The risk of bias in each included study will be assessed utilizing the Cochrane Collaboration's risk of bias tool. Two researchers will independently evaluate the bias based on the following items: random sequence generation, allocation concealment, blinding of the participants and personnel, blinding of the outcome assessments, incomplete outcome data, selective reporting, and other sources of bias. The studies will be evaluated as low risk, high risk, and unclear risk. Inconsistencies will be resolved by discussion with other reviewers.

#### 2.3.4 Data analysis

The Review Manager 5.3 (Cochrane Collaboration, Oxford, UK) will be used to analyze the data. For outcomes, we will use relative risk (RR) and 95% confidence interval (CIs) to evaluate dichotomous outcomes, while using standardized mean difference (SMD) and mean difference (MD) with 95% CIs to assess continuous variables. The heterogeneity between RCTs will be calculated by Cochrane *X*^*2*^ and *I*^*2*^ tests.^[11]^ If *P* ≥ .05 and *I*^2^ ≤ 50%, no statistical heterogeneity is observed, the data will be calculated with a fixed-effect model. If *P* < .05 and *I*^*2*^ > 50%, the random effect model will be used.

#### 2.3.5 Subgroup analysis

If there is significant heterogeneity, subgroup analysis will be conducted based on different interventions, controls, durations of treatment, and outcome measures.

#### 2.3.6 Sensitivity analysis

We will carry out sensitivity analyses to investigate the robustness of the study conclusions. In this way, we will be able to assess the impact of low-quality studies on the overall results and whether the results are robust.

#### 2.3.7 Assessment of publication biases

If there are more than 10 studies included, a funnel plot analysis will be drawn to assess the publication bias and Egger test in Stata 14.0 (Stata Corp, College Station, TX) will be conducted for statistical investigation.

#### 2.3.8 Assessment of quality of evidence

We will use the Grading of Recommendations Assessment, Development, and Evaluation (GRADE) to assess the results. In the GRADE system, the quality of evidence will be categorized into 4 levels: high, moderate, low, and very low quality.

#### 2.3.9 Ethics and dissemination

This systematic review will not require ethical approval because there are no data used in our study that are linked to individual patient data. The results of this systematic review will be disseminated only in a peer reviewed publication.

## 3 Discussion

As the pace of population aging accelerates in China, the incidence of CI is increasing rapidly, imposing a major economic burden on the family and society.^[12,13]^ According to the theory of Chinese medicine, “blood stasis and stagnation” is regarded as the core pathogenesis of CI.^[14]^ Therefore, promoting blood circulation and removing blood stasis is a vital method for the treatment of CI.^[15]^ SXT injection, a purified extract of *leech* and *earthworm*, is used to treat the patients of “blood stasis and stagnation”.^[16]^ Studies have shown that SXT can alleviate the injury of nerve function, improve the blood lipid metabolism and coagulation function in patients with CI.^[17,18]^ Currently, there is no systematic review to address the efficacy and safety of SXT for the treatment of CI. Therefore, we will conduct a systematic review and meta-analysis of RCT to evaluate the efficacy of SXT in the treatment of CI. We hope that the result of this review will provide more reliable references to help clinicians make decisions when dealing with CI.

### 3.1 Amendments

If amendments are needed, we will update our protocol to include any changes in the whole process of research.

## Author contributions

**Conceptualization:** Shangwei Hu.

**Data curation:** Hua Fang, Honglong Zhou, Jicai Zhang, and Ziyi Li.

**Formal analysis:** Zhen Chen, RaoRao Yuan, Xiangqun Huang, and Junyong Yang.

**Methodology:** Hua Fang and Honglong Zhou.

**Software:** Hua Fang and Honglong Zhou.

**Supervision:** Jianqiang Zhang, Shuo Wang, and Yong Huang.

**Visualization:** Hua Fang and Honglong Zhou.

**Writing – original draft:** Hua Fang and Honglong Zhou.

**Writing – review & editing:** Shangwei Hu.
